# Serial Electrocardiographic Changes in Early Post-myocardial Infarction Pericarditis

**DOI:** 10.7759/cureus.26795

**Published:** 2022-07-12

**Authors:** Kazuhito Hirata, Masanori Kakazu, Tomohiro Arakaki, Atsushi Kakazu, Osamu Arasaki

**Affiliations:** 1 Cardiology, YuuAi Medical Center, Tomigusuku, JPN

**Keywords:** phonocardiography, electrocardiography (ecg), friction rub, myocardial infarction, acute pericarditis

## Abstract

A 72-year-old man developed fever and chest pain, accompanied by an increase in C-reactive protein, four days after successful emergency catheter intervention for an acute wide anterior myocardial infarction (MI). A twelve-lead electrocardiogram (ECG) showed marked ST elevation in leads V1-6, I, and aVL, with reciprocal ST depression in leads II, III, and aVF. Although these ECG changes improved by day three, he developed fever and chest pain on day four, and an ECG at this timepoint showed ST elevation in leads II, III, aVF, and mild worsening of the ST elevation in the anterolateral leads, indicating diffuse ST-segment elevation consistent with acute pericarditis. Despite the presence of a typical friction rub, there was no pericardial effusion on an echocardiogram. No elevation of cardiac enzymes was noted. A diagnosis of early post-infarction pericarditis was made, and the patient was successfully treated with acetaminophen and colchicine. Early post-infarction pericarditis (EPIP), albeit rare in the era of emergency catheter treatment, is important because it may indicate a large transmural infarction and must be differentiated from re-infarction. Fever, chest pain, friction rub, ST elevation in the leads distant from the infarct area, recurrence of ST-segment elevation in the infarct area, and increase in inflammatory markers but not cardiac enzymes were crucial for establishing a diagnosis of EPIP.

## Introduction

Early post-infarction pericarditis (EPIP) is caused by inflammation of the injured myocardium and usually reflects a large infarct size [1]. However, electrocardiographic (ECG) changes due to EPIP, and recurrent myocardial infarction (MI) are sometimes difficult to distinguish [2,3]. Here we describe a case of EPIP wherein detection of bedside friction rub and diffuse but transient ST elevation in the ECG were important for arriving at an accurate diagnosis.

## Case presentation

**Figure 1:**
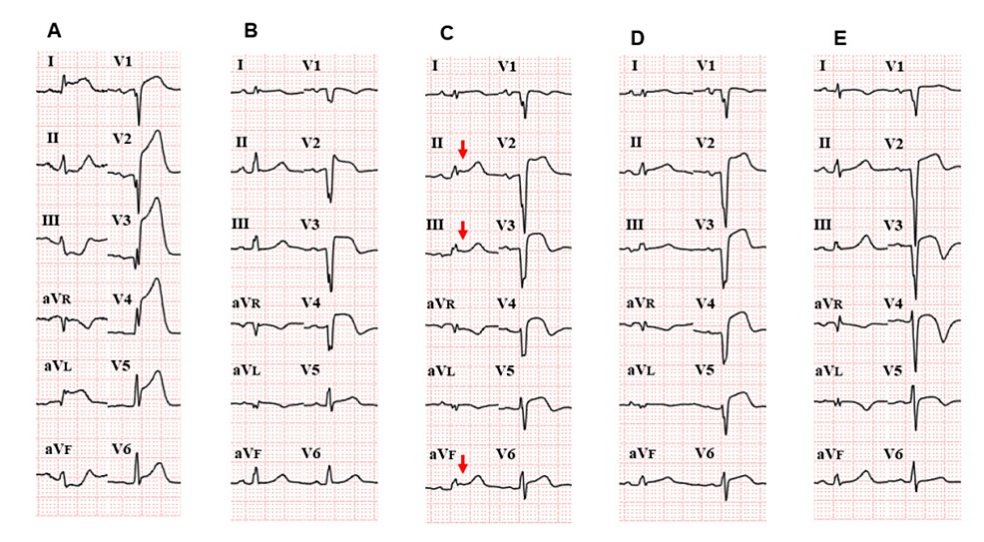
Serial twelve-lead electrocardiograms A) Day one: initial ECG shows wide anterior myocardial infarction (MI) with ST-segment elevation in V1-V6, I, and aVL, along with reciprocal ST depression in leads II, III, and aVF. B) Day three: ECG changes improved after catheterization of the occluded proximal left anterior descending artery. C) Day four: ECG revealed ST-segment elevation (arrows) and PR segment depression in leads II, III, and aVF. ST-segment elevation in the anterior precordial leads had worsened, suggesting diffuse ST elevation consistent with acute pericarditis. D) Day five: ST elevation in the inferior lead started to resolve and the previously inverted T waves in V3-V5 temporarily returned to baseline. E) Day nine: ST elevation in the inferior leads returned to baseline with improvement in ST elevation; however, T wave inversion had recurred.

A 72-year-old male presented with chest pain. Initial vital signs were blood pressure of 139/84 mmHg, heart rate of 70/min, respiratory rate of 20/min, and SpO2 of 98% on ambient air. Initial ECG revealed marked ST-segment elevation in leads V1-6, I, and aVL, and ST depression in leads II, III, and aVF, consistent with acute wide anterior MI (Figure 1A).

Emergency coronary angiography revealed total occlusion of the proximal left anterior descending artery at the ostium, which was successfully resolved by emergency percutaneous coronary intervention and stent placement. The patient’s clinical course was complicated by mild congestive heart failure that was responsive to intravenous carperitide (human atrial natriuretic peptide: 0.0125 microgram/kg/min for one day) and furosemide 20 mg twice a day for four days, followed by oral azosemide 30 mg and spironolactone 25 mg daily. An echocardiogram showed antero-apical hypokinesis with an ejection fraction of 40%. The patient remained stable up to day three, with improvement in ST-segment elevation (Figure 1B).

**Figure 2:**
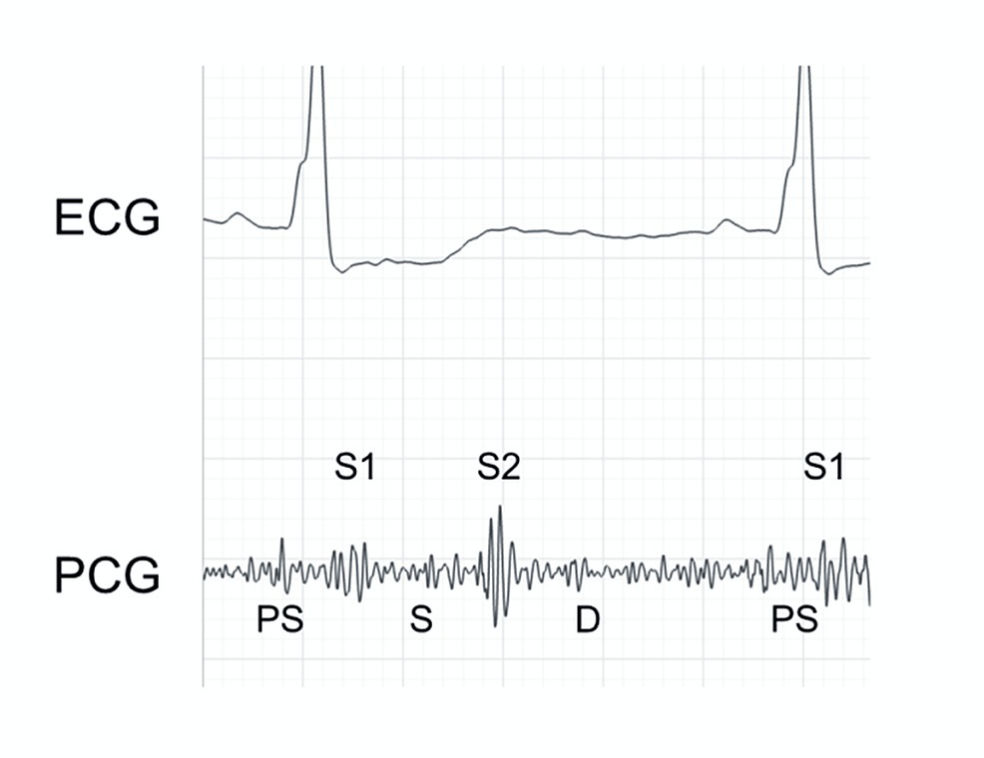
A phonocardiogram obtained on day three A phonocardiogram obtained on day three at the bedside at the left fourth interspace with the patient in the supine position. Typical friction rub with presystolic (PS), systolic (S), and early diastolic (D) components were recorded with an electric stethoscope (Eko Devices, Inc. Oakland, California).

On day four, the patient complained of mild chest discomfort, and auscultation of the heart revealed a typical friction rub with triphasic scratching components at the fourth interspace (Figure 2, Video 1).

**Video 1:** Supplemental video for friction rub Friction rub recorded at bed side with an electronic stethoscope Eko Duo. It is recommended to use earphones or headphones with the volume turned up.

He also developed a fever of 38.5°C and raised C-reactive protein (CRP) levels (14 mg/dl: normal <0.14mg/dl). A repeated ECG at this time revealed ST elevation and PR depression in leads II, III, and aVF, in addition to mild worsening of ST elevation in the anterior precordial leads (Figure 1C). No elevation in cardiac enzymes was seen. Echocardiography showed anteroseptal wall hypokinesis with an ejection fraction of 40% (no change compared with the initial echocardiogram) without pericardial effusion. Based on the symptoms, ECG findings, and typical friction rub, acute EPIP was diagnosed. Because the chest pain was mild and transient, the hemodynamic condition was stable, and the ECG change was subtle, emergency coronary angiography was deferred. The patient was prescribed colchicine 0.5 mg once a day, and acetaminophen 500 mg was given only when his temperature rose up to 38.5°C.

The fever subsided by day five, and his vital signs remained stable throughout. Subsequently, the degree of ST elevation and PR segment depression in the inferior leads improved by day five (Figure 1D), and T wave inversion in the anterolateral leads temporarily returned to baseline. ST elevation in the inferior leads returned to baseline by day nine (Figure 1E). In the anterolateral leads, the degree of ST elevation had improved, T wave inversion was again seen. Despite improvement in symptoms, ECG findings, and CRP levels (0.38mg/dl), the friction rub continued to be heard till day nine (day of discharge). The patient was discharged home with aspirin 100 mg, prasugrel 3.75 mg, azosemide 30 mg, spironolactone 25 mg, bisoprolol 2.5 mg, losartan 12.5 mg, and colchicine 0.5 mg once a day. The friction rub had disappeared when he arrived for his first outpatient clinic follow-up, which was two weeks later. The patient had follow-up coronary angiography one year later, which did not show re-stenosis in the stent placed in the proximal left anterior descending artery. 

## Discussion

There are two types of post-MI pericarditis, namely, EPIP, as seen in our patient, and Dressler syndrome, i.e., late post-MI pericarditis [1]. Both types of pericarditis are due to an immune response triggered by myocardial damage and an accumulation of debris or blood in the pericardium, which are associated with the infarct size [1, 4-6]. Usually, EPIP occurs within three to four days after the onset of MI with symptoms of chest pain (80%-85%), low-grade fever, friction rub (50%), and elevated inflammatory markers such as C-reactive protein [1, 4, 6].

The incidence of pericarditis or pericardial effusion after MI has been reported to be 10%-20% in the pre-thrombolysis era [1, 4-6], and those with moderate pericardial effusion showed a higher incidence of 30-day mortality and free wall rupture [5]. However, early catheter-based interventional therapy has reduced the incidence of EPIP and the Dressler syndrome to 4% and 0.5%, respectively, presumably due to a smaller infarct size [1, 4, 6]. However, the diagnosis of post-MI pericarditis based on conventional criteria lacks both sensitivity and specificity and may underestimate the actual incidence [1, 6, 7].

Cardiac MRI is a useful tool for identifying MI and visualizing pericardial effusion and inflammation. Doulaptsis et al. have used MRI with late gadolinium enhancement at two to five days after infarction to evaluate the incidence of EPIP among 189 consecutive patients with ST-elevated MI (STEMI) who underwent primary catheter intervention [7], and they reported that 16.9% (32/189) of patients had pericardial effusion while 31% (58/189) had pericardial inflammation. Further, such pericardial inflammation was mostly localized to the area of myocardial injury (60.3%, 35/58) even though inflammation had spread diffusely (27.5%, 16/58) [7]. In our patient, ST-segment elevation and PR depression were prominent in the inferior leads, and ST-segment re-elevation was seen in the anterolateral leads, indicating diffuse pericardial inflammation. If EPIP was localized to the area of infarction, such as in 60% of cases in the cohort of Doulaptsis, only ST-segment re-elevation and delayed inversion of the T wave in the infarct area may have been seen, which would have rendered differentiation from re-infarction challenging [3].

Usually, EPIP is self-limiting and does not require specific treatment if the effusion is small and the patient is hemodynamically stable. Our patient was treated with colchicine and acetaminophen to control clinical symptoms due to inflammation and to prevent the recurrence of pericarditis. However, larger effusions may be an early sign of oozing rupture and, therefore, require attention as progression to tamponade is possible [5].

## Conclusions

An accurate diagnosis of EPIP is important because it is associated with a larger infarct size and may mimic re-infarction. Further, as even moderate pericardial effusion may be a harbinger of subsequent free wall rupture, physicians treating patients in the acute phase of MI should be aware of EPIP because cognate ECG changes might be subtle and transient, as seen in our case.
